# Memoir upon the Organs of Absorption in Mammiferous Animals, Read at the Institute, the Seventh of August, 1809

**Published:** 1811-01

**Authors:** M. Magendie

**Affiliations:** Doctor in Medicine of the Faculty of Paris


*For the Medical and Physical Journal.*

### *Memoir upon the Organs of Absorption in Mammiferous Animals, read at the Institute, the seventh of August,* 1809. By M. MAGENDIE, Doctor in Medicine of the Faculty of Paris.

AMONGST the facts which I have had the honour of relating to the class, in a memoir upon the Upas, nux vomica, and St. Ignatius's bean,* one in particular seems worthy of attention; I allude to the facility with which these poisonous substances are absorbed and introduced into the sanguineous system. Scarcely twenty seconds being necessary for them to pass from the peritoneal cavity to the spinal marrow.

The ideas generally received respecting the organs of absorption, leave no doubt that the lymphatic vessels are the agents which convey these poisons into the circulating system. Thus, in the experiment, where poison is inserted in the thigh of an animal, there are not two modes of explaining the mode of its absorption. We must admit, that it is taken up from the wound by the lymphatic vessels of the

* Faba Sanct. Ignat. is a species of the Genus Ignatia, and is the I. Amara. Pentandria Monogynia.

Jussieu confounds the Genus Ignatia with the genus Strychnos, and probably this author may have done the same. In that case the Strychnos potatorum, the seed of which has the remarkable property of purifying water, and is used for that purpose throughout the East, will be, perhaps, the *Faba S. I.* The synom. of the S. potatorum, are

S. Tentankotta.
S. Titou-cote.

parts parts with which it was in contact; that after being absorbed, it is conveyed by these vessels to the inguinal glands, and thence, still by lymphatic vessels, to the thoracic duct; and is ultimately introduced into the sanguineous system, by the communication between that canal and the subclavian veins.

The experiments which I am about to detail, were instituted with the design of adding a degree of certainty to the explanation of these facts, at present, generally admitted, and our labour only took a particular direction when a great number of facts obliged us to modify our views of the subject. An absorption so rapid, by vessels characterized by weakness and slowness of action; a poisonous matter readily passing along the tortuous and difficult course of the lymphatic glands without being subjected to any alteration, were facts calculated to throw some doubt on the correctness of the received opinion. But this explanation is given by so many respectable men, and is supported by experiments so decisive, that, even now, when we have several facts to oppose to it, we dare not assert that it is incorrect, only, that it is not admissible in all circumstances.

Before we give an exposition of our own experiments, it may not be useless to recall to notice an hypothesis, which for some time rendered the received opinion on the organs of absorption doubtful.

This hypothesis, professed by Boerhaave, Haller, Meckel, Riusch, Swammerdam, and others, consisted in supposing that the veins divide the absorbing faculty with the lymphatic vessels. It is supported by various circumstances of structure, and some physiological and pathological facts. A series of interesting experiments, enterprized and executed a few years since at the veterinary school of Alfort, has given a yet further degree of probability to the absorbing powei of the veins, without, however, being of a nature to produce complete conviction. An opinion founded on the physical disposition of the organs, upon experiments sufficiently numerous and conclusive, and supported by the names of Boerhaave, of Ruisch, and of Haller, ought not to be readily abandoned. Nothing short of the anatomical discoveries of the last century, on lymphatic vessels; the admirable experiments of the two Hunters, of Cruickshanks, of Mascagni, of Degenettes, could have established the position, that the lymphatic vessels alone possessed the faculty of absorbing.

In support of the general opinion, may also be cited the very curious experiments recently made by M. Dupuytren. This physiologist tied the thoracic duct in several horses; some of them died in five and six days, others preserved all the the appearances of health. It was already known by an experiment of Duverney, by some observations on an obstructed thoracic duct, and especially, by the experiments of Flandrin, that the thoracic duct might cease to pour chyle into the subclavian vein, without the death of the animal being occasioned. It was known, indeed, that certain animals had died in consequence of a ligature round the duct, but the cause of this diversity of result was not understood. By his experiments, M. Dupuytren has ascertained it in a very satisfactory manner. In those animals that died in five or six days after the thoracic duct was tied, it was always impossible to make an injection pass from the inferior portion of the canal into the subclavian vein; consequently it is probable that as soon as the ligature was tied, the chyle ceased to flow into the venous system. On the contrary, in those animals which survived the ligature, it was always easy to make any fluid pass from the lower part of the duct into the subclavian veins, by means of very numerous communications established between them, by lymphatic vessels situated in the posterior as well as in the anterior mediastinum. I was present at the opening of a horse, in which M. Dupuytren had tied the thoracic duct upwards of six weeks before, and I was readily convinced, that evident communications existed between the inferior portion of the canal, aud the subclavian veins, although the canal was entirely obliterated where the ligature was tied.

I shall now state the experiments which I have made, chiefly in concert with M. Delille, to determine whether the lymphatic vessels really afford the only passage by which foreign bodies enter the venous system.

Experiments upon absorption have always been attended with a degree of obscurity, from the difficulty of demonstrating the passage and the presence of the absorbed substances, either in the lymphatic vessels, or in the blood vessels. In employing the upas, or nux vomica, we have not to fear these inconveniences; it is well known that two *centigrammes**, of these substances produce effects too remarkable to be disputed.

The first question we proposed to resolve, was—Will a ligature round the thoracic duct prevent poison from passing into the sanguineous system, and in course, its effects on the spinal marrow?

I passed a ligature round the thoracic duct of a dog, a little before the opening into the left subclavian vein; and im-

* Centigramme. E. grain .1544.

mediately mediately introduced a solution of upas into the cavity of the peritoneum. The effects of the poison were as speedy and as marked, as if the duct had not been tied. I tied a ligature upon other animals in the same manner; but instead of introducing the poison into the cavity of the peritoneum, I inserted it in the pleura, in the stomach, in the intestines, in the femoral muscles, &c. The effects, in every instance, were as rapid and as fatal as if the duct had been free.

We cannot draw very certain conclusions from these first essays; for we know that the thoracic duct is not the only point of communication between the lymphatic system and the venous system. There is usually a second thoracic duct on the right side, almost as considerable as that on the left side; large lymphatic trunks frequently open separately into the subclavian veins; and the lacteal canal, still more frequently, has several openings into the vein, in which it terminates. One of these circumstances was probably the case with the animals subjected to our experiments; it was therefore necessary to make further attempts from which less doubtful results might be obtained.

We made an incision through the abdominal parietes of a dog, which had eaten a large quantity of food seven hours before, that the lymphatic vessels of the abdomen might be easy to distinguish, and we drew out a fold of small intestine, round which we applied two ligatures, at four decimetres * from each other. The lymphatics on this fold of intestine were very white, and very apparent from the chyle which they contained. Two ligatures were tied on each of these lymphatics at the distance of a centimetre; we then cut the vessels between the ligatures. This part of the experiment was conducted with great care, and we assured ourselves, by all possible means, that the convolution of intestine out of the abdomen had no communication by the lymphatics with the rest of the body. Five arteries and five mesenteric veins were included in the fold of intestine between the two ligatures; four of these arteries and veins were tied and cut in the same manner as the lymphatics; the two extremities of our fold of intestine were cut and entirely separated from the rest of the small intestine. Thus we had a portion of intestine four decimetres long, communicating with the rest of the body only by one artery and one mesenteric vein; these two vessels were isolated in length about the space of three inches; we even stripped off the cellular tunic, lest any lymphatics should remain concealed in it.

| | E. inches. |
|---|---|
| * Decimetre | 3,93702 |

To

To obtain a positive result, nothing more was requisite than to inject a small quantity of upas into the cavity of the fold of intestine. This was executed with proper precaution, to prevent the injected liquid escaping. The fold of intestine, enveloped in a piece of fine linen, was replaced in the abdomen; it was then exactly one o'clock. To our great astonishment, at six minutes past one, the general effects of the poison appeared with their ordinary intensity, in as great a degree, indeed, as if the fold of intestine had been in its natural state.

The animal being dead, we examined the state of the parts; none of the ligatures were displaced; nothing excited any suspicion that the poison had passed into the cavity of the abdomen.

This experiment, repeated several times without the least variation in the result, appeared to us very positive; it proves at least as much as can be proved by physiology, that the lacteal vessels are not exclusively the organs of intestinal absorption.

This species of absorption, different from lymphatic absorption, might be peculiar to the intestine; it was therefore important to ascertain whether it obtained in other parts of the body.

We separated from the body the thigh of a dog, previously stupified by opium (to spare him the pain of a difficult experiment); the division of the thigh was effected in a manner, that it still communicated with the trunk by the crural artery and vein. We took the same precautions with respect to these two vessels, as for the mesenteric vein and artery in the preceding experiment; *i. e.* we isolated them for an extent of four decimetres, and stripped off the cellular coat. We then inserted two grains of poison in the paw of the animal, and watched the event.—The poison produced its effects as rapidly and as powerfully as if the thigh had not been separated from the body; so much so that the first signs of the upas operating, were manifested before the fourth minute, and before the tenth, the animal was dead.

It might be objected that, notwithstanding all our precautions, the coats of the veins and arteries might still contain lymphatics, and that these vessels were sufficient to afford a passage for the poison. It was easy to remove this objection.

I repeated the preceding experiment on another dog, with this modification: I introduced into the crural artery a small quill, upon which I fastened the vessel by two ligatures: the artery was then divided between the two strings. The crural vein was treated in the same manner; so that there there was no communication between the thigh and the rest of the body, except by the arterial blood which was brought to the thigh, and the venous blood which returned to the trunk. The poison being inserted in the paw, produced its general effects in the usual time, that is, in about four minutes.—From these different experiments, I think we may conclude, that the lymphatic system is not, at least in certain cases, the exclusive way by which foreign substances pass into the venous system.

This new mode of absorption, much more direct than lymphatic absorption, enables us to conceive the rapidity with which various deleterious, or other matters, are absorbed, as well as the promptitude with which their effects are produced on the system.

But what are the organs which take up the poison in the parts where it has been introduced? Are they venous extremities, or are they lymphatic capillaries, which having immediate anastomoses with the sanguineous capillaries, might thence convey the poison into the venous system?

The experiments which I have just stated, added to all those which have been attempted on the same subject, appear to me wholly insufficient to determine either of these questions; only, it ought to be remarked, that our experiments strongly support the opinion that there is a direct absorption by the veins.

But there is a fact rendered evident by the preceding experiments, upon which it is necessary to dwell for a moment; it is, that the venous blood is charged with the poison, and that through the medium of this blood, the poison is enabled to produce its deleterious action upon the organs. In effect, if in the experiments in which I had separated the thigh from the trunk, we were to suspend the course of the venous blood by pressing the crural vein between two fingers, we should diminish, and even totally prevent the production of bad consequences. The blood of an animal, then, in which signs of the action of the upas are manifested, contains a portion of poisonous matter; we may say, that such blood is really poisoned. It was curious and interesting to know if this blood, conveyed into the circulating system of a sound animal, would produce effects similar to those which it occasions in the animal in which it was originally inserted. At first, we are disposed to think this is very probable, even that it is certain. The following experiments will shew how careful we ought to be in physiological inquiries to distinguish that which is probable, from that which is proved by experience.

We caused the arterial blood of an animal, in which the tetanos, occasioned by the upas, was evident, to pass into the jugular vein of a sound animal. The transfusion lasted nearly twenty minutes, so that the sound animal received a considerable quantity of poisoned blood, blood which in the first moment of the experiment was red and vermillion, and which at length became violet, and black, when the upas had produced asphyxy. Yet no appearance of irritation in the spinal marrow was evinced, and the animal suffered nothing more than what happens in ordinary transfusions conducted with every precaution. He had during some hours, a marked acceleration of the inspiratory and expiratory actions, and an abundant pulmonary exhalation.—The experiment was repeated several times, and always gave the same results.

After this we were certain that the arterial blood of animals poisoned by the upas, nux vomica, or St. Ignatius's bean, was not capable of producing ill consequences in other animals: perhaps it might be different in venous blood. It might be presumed that the act of respiration changed the nature of the poisonous substance; and that this change might give, to a certain extent, the reason why the transfusion of the blood of animals poisoned by the strychnos, is not followed by any bad consequences.

This circumstance did not hold with regard to the venous blood, which returns from the part where the poison has been introduced. After the experiment which we have related upon the upas, it is impossible to doubt that the poison is conveyed to the lungs by the venous blood. It was very probable that this blood introduced into the system of another animal, would produce accidents similar to those which it occasioned in the animal which was inoculated with the poison.

A small piece of wood covered with two grains of upas was inserted in the left side of the snout of a dog.—Three minutes afterwards, we caused the blood to pass from the jugular vein of the side in which the poison had been introduced, into the venous system of another dog. The transfusion was began about a minute before the first signs of the upas having taken effect were apparent, and ended only with the death of the animal which experienced them. No appearance of irritation in the spinal marrow was perceived in the dog which had received so much poisoned blood.

The experiment was repeated several times with variations in the mode of introducing the poison; but we never could perceive any thing which resembled the effects of the action of the strychnos in the sound animals which had suffered the transfusion of the poisoned blood.

These

These decisive results seem sufficient to permit us to conclude, that the venous blood of animals poisoned by the upas, nux vomica, and St. Ignatius's bean, is as incapable as the arterial blood of producing in another animal the ill consequences which they would occasion in the animal to which they had been immediately applied.

If doubts still remained, the following experiment, several times repeated, would set the question at rest.

We separated the thigh from the body of an animal, as in the preceding experiment, isolating the crural artery and vein; we introduced the poison into the paw of the divided limb, and transfused the blood from the crural vein into the jugular vein of a sound animal. The passing of the blood from one animal to the other lasted above six minutes, being much more than sufficient time for the upas to produce its effects; yet this poison gave no indication of having acted.—We must not, however, believe that in this experiment the transfused blood, from some particular cause, had no deleterious properties: the experiment which we are about to detail proves the contrary.

As in the last experiments, I separated the thigh from the body; three minutes after having introduced poison into the paw, I made the blood pass from the crural vein into the jugular vein of another animal; the transfusion was continued five minutes without occasioning bad consequences; I then stopped it, and caused the blood of the crural vein to return to the animal to which it belonged. This animal almost immediately presented evident signs of the action of the strychnos on the spinal marrow.

From the several experiments related in this memoir, I think we may conclude,

1. That the lymphatic vessels are not always the channel through which foreign substances pass to the sanguineous system.

2. That the blood of animals upon which the bitter strychnos produce their deleterious effects, cannot produce fatal accidents in other animals.

I think it would be premature at present, to offer any explanation of this singular phenomenon. In physiological science we ought to be sparing in conjectures, and prodigal in facts.

